# Clinical significance of lncRNA-ATB expression in human hepatocellular carcinoma

**DOI:** 10.18632/oncotarget.21094

**Published:** 2017-09-20

**Authors:** Se Young Jang, Gyeonghwa Kim, Soo Young Park, Yu Rim Lee, Sang Hoon Kwon, Hyeong Seok Kim, Jun Sik Yoon, Jun Seob Lee, Young-Oh Kweon, Heon Tak Ha, Jae Min Chun, Young Seok Han, Won Kee Lee, Jun Young Chang, Jung Gil Park, Byungheon Lee, Won Young Tak, Keun Hur

**Affiliations:** ^1^ Department of Internal Medicine, Kyungpook National University School of Medicine, Daegu, Republic of Korea; ^2^ Department of Biochemistry and Cell Biology, Cell and Matrix Research Institute, School of Medicine, Kyungpook National University, Daegu, Republic of Korea; ^3^ Department of Surgery, Kyungpook National University School of Medicine, Daegu, Republic of Korea; ^4^ Biostatistics, Medical Research Collaboration Center in Kyungpook National University Hospital, Kyungpook National University School of Medicine, Daegu, Republic of Korea; ^5^ Department of Neurology, Gyeongsang National University Changwon Hospital, Changwon, Republic of Korea; ^6^ Department of Internal Medicine, College of Medicine, Yeungnam University, Daegu, Republic of Korea

**Keywords:** hepatocellular carcinoma, long noncoding RNA ATB, survival, prognosis, epithelial mesenchymal transition

## Abstract

Hepatocellular carcinoma (HCC) is a worldwide health problem and it is important to understand the mechanistic roles of the biomolecules involved in its pathogenesis. Long non-coding RNAs (lncRNAs) are frequently and aberrantly expressed in various human cancers and are known to play a role in cancer pathogenesis. The aim of this study was to analyze the expression of lncRNA-ATB in HCC and investigate the implications for prognoses.

In total, 100 samples of HCC tissues and their corresponding, adjacent, non-cancerous liver tissues were collected. Total RNAs were extracted and the expression levels of lncRNA-ATB were measured by qRT-PCR. The association of lncRNA expression with clinicopathological features and patient survival were then analyzed.

LncRNA-ATB was significantly upregulated in HCC tissues compared with the levels in corresponding non-cancerous tissues. Expression of lncRNA-ATB was significantly associated with portal vein thrombosis, intrahepatic or extrahepatic metastases, mUICC stage, and the BCLC stage. Large tumors (> 5 cm, HR = 3.851, 95% CI = 1.431–10.364, *p* = 0.008) and higher lncRNA-ATB expression (HR = 4.158, 95% CI = 1.226–14.107, *p* = 0.022) were the significant prognostic factors for overall survival.

With this novel evidence of the involvement of lncRNA-ATB in HCC pathogenesis and clinical features, lncRNA-ATB can be concluded to have potential as a biomarker for the prognosis of HCC and as a targeted therapy for afflicted patients.

## INTRODUCTION

Hepatocellular carcinoma (HCC) is one of the leading causes of cancer-related deaths worldwide [1] due to the challenges encountered with its treatment. In many cases, HCC is diagnosed at an advanced stage, which limits treatment options and affects prognosis [2]. Moreover, HCC shows a variety of clinical results due to the etiology of liver disease, underlying liver functions, and tumor biology, even for tumors at the same cancer stage. Unfortunately, alpha-fetoprotein (AFP), which currently is used widely as a biomarker for HCC, lacks specificity for HCC screening and diagnosis [3]. Other biomarkers, such as des-gamma carboxyprothrombin and fucosylated AFP, have been investigated for their clinical efficacy but have also shown low accuracy [4]. Recently, due to tumor molecular heterogeneities of HCC that were found to result in outcome differences, non-coding RNAs and microRNAs are now considered to be novel potential prognostic biomarkers. Therefore, it is necessary to find a novel biomarker for prognostic prediction that in addition to HCC diagnosis and surveillance can enable curative treatment of the disease and improve patient survival.

The development of RNA sequencing technologies has led to the discovery of a wide range of non-coding RNA genes, including long non-coding RNAs (lncRNAs), broadly defined as endogenous cellular non-coding RNA molecules that are longer than 200 nucleotides. LncRNAs perform multiple functions, such as signals, decoys, guides, and scaffolds [5, 6]. As indicated by many studies, lncRNAs are frequently and aberrantly expressed in various human cancers, having potential roles as both oncogenes and tumor suppressors [7]. In addition, recent studies have suggested that lncRNAs could be potential prognostic biomarkers for multiple cancers [8–10].

The lncRNA activated by tumor growth factor-beta (TGF-β) (LncRNA-ATB) and lncRNA maternally expressed gene 3 (lncRNA-MEG3) are known to function as an oncogene and a tumor suppressor gene, respectively. One study showed that lncRNA-ATB might be involved in the progression of colorectal cancer and could be a novel indicator of poor prognosis in afflicted patients [11]. Another study demonstrated that lncRNA-ATB plays an important role in the epithelial–mesenchymal transition (EMT) by promoting invasion and metastasis through the TGF-β/microRNA 200 (miR-200)/zinc finger E-box-binding homeobox (ZEB) axis, resulting in poor prognoses for gastric cancer [12]. Recently, lncRNA-ATB was shown to induce EMT, promote tumor cell invasion, and play a key role in distant metastasis of HCC [13]. EMT converts cancerous epithelial cells to mesenchymal-like cells by giving them migratory and invasive properties, which enables primary tumor cells to move, colonize distant organs, and form secondary tumors. EMT is known to be regulated by a complex signaling network involving both transcriptional and post-transcriptional regulatory pathways.

LncRNA-MEG3, which is part of the *DLK1-MEG3* locus located on chromosome 14q32 [14], is usually either normally or highly expressed in various tissues, including liver [15], brain and meninges [16], lung [17], and bladder [18]. However, its expression is downregulated in many cancers [18–22], including HCC [23], where it is predicted to act as a tumor suppressor in the pathogenesis and progression of cancers. According to a previous study, lncRNA-MEG3 is considered to be involved in p53 regulation and has a concomitant effect on cell survival and proliferation [24].

Therefore, we carried out this study to assess the possibility that lncRNAs could serve as biomarkers for HCC prognostic prediction. We observed the expression levels of lncRNA-ATB and lncRNA-MEG3 in tissue samples from HCC patients and analyzed their correlations with clinicopathological features, disease recurrence, and survival of HCC patients.

## RESULTS

### Overexpression of lncRNA-ATB and lncRNA-MEG3 in HCC tissues

**Figure 1:**
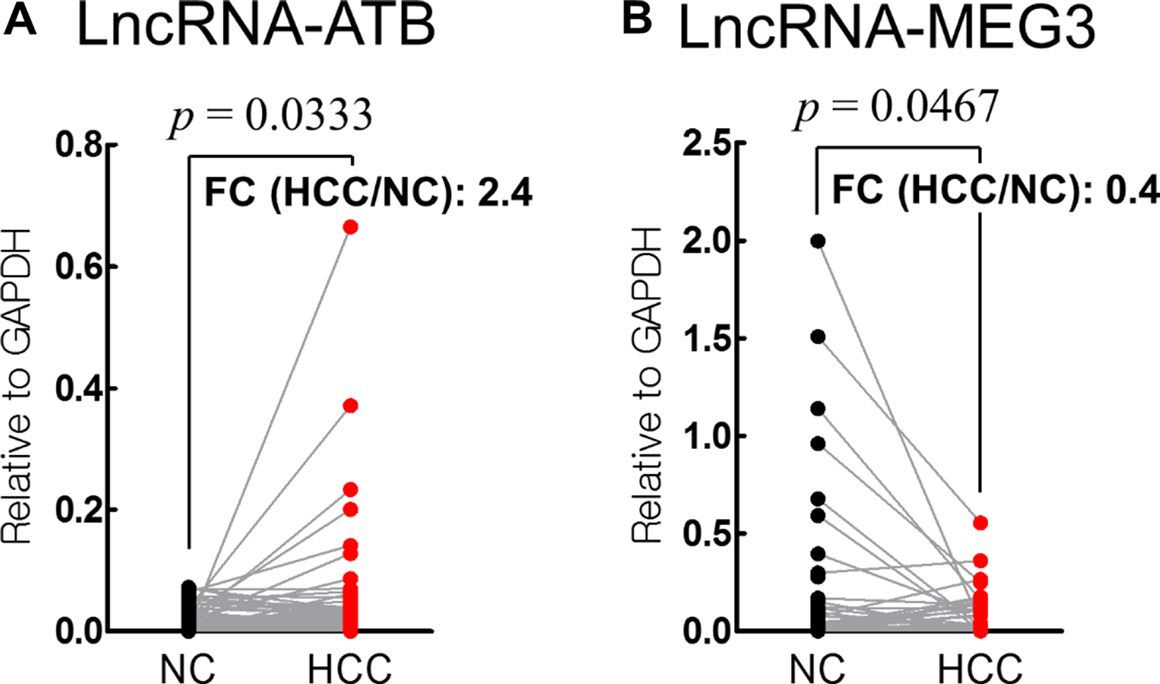
Analysis of lncRNA-ATB (**A**) and lncRNA-MEG3 (**B**) in HCC and corresponding non-cancerous (NC) tissues. (FC: fold change).

LncRNA-ATB expression was significantly upregulated in HCC tissues (fold change = 2.4; *p* = 0.033), whereas that of lncRNA-MEG3 was down-regulated (fold change = 0.4; *p* = 0.047), compared with the levels in the corresponding non-cancerous liver tissues (Figure 1).

### Baseline characteristics of 100 HCC patients

As shown in Table 1, the mean age of the patients was 60 years (range: 32–88 years), and the majority were male (*n* = 88, 88.0%). The Child-Turcotte-Pugh (CTP) classes were class A for 80 (80.0%) patients and class B for 20 (20.0%) patients. The etiologies of liver disease were viral hepatitis (*n* = 78, 78.0%), alcohol (*n* = 16, 16.0%), and cryptogenic (*n* = 6, 6.0%). Portal vein thrombosis was observed in 29 (29.0%) patients. Forty-seven cases of HCC were stage I or II and 53 cases were stage III or IV, according to the modified Union for International Cancer Control (mUICC) stages.

**Table 1:** Baseline characteristics of the 100 HCC patients

Clinical characteristics	Total (*n =* 100)
Age (years)	60.2 ± 11.5
Gender	
male	88 (88.0%)
female	12 (12.0%)
Etiology	
HBV	68 (68.0%)
HCV	9 ( 9.0%)
Alcohol	16 (16.0%)
HBV & HCV	1 (1.0%)
Cryptogenic	6 ( 6.0%)
Number of tumors	
Single	52 (52.0%)
Multiple	48 (48.0%)
Size of tumor	
≤ 5 cm	54 (54.0%)
> 5 cm	46 (46.0%)
PVT	
no	71 (71.0%)
yes	29 (29.0%)
Modified UICC stage	
I–II	47 (47.0%)
III–IV	53 (53.0%)
BCLC stage	
0–A	41 (41.0%)
B–C	59 (59.0%)
CTP class	
A	80 (80.0%)
B	20 (20.0%)
AST (U/L)	45.0 (27.5–73.5)
ALT (U/L)	32.0 (23.5–47.5)
Bilirubin (mg/dL)	0.8 (0.5–1.3)
Albumin (g/dL)	3.9 (3.3–4.1)
Prothrombin time (sec)	12.3 (11.5–13.6)
AFP (ng/mL)	21.1 (5.3–486.3)

Note: HBV, hepatitis B virus; HCV, hepatitis C virus; PVT, portal vein thrombosis; ALT, alanine transaminase; AST, aspartate transaminase.

### Correlation between lncRNA-ATB expression and clinical features in HCC patients

**Figure 2:**
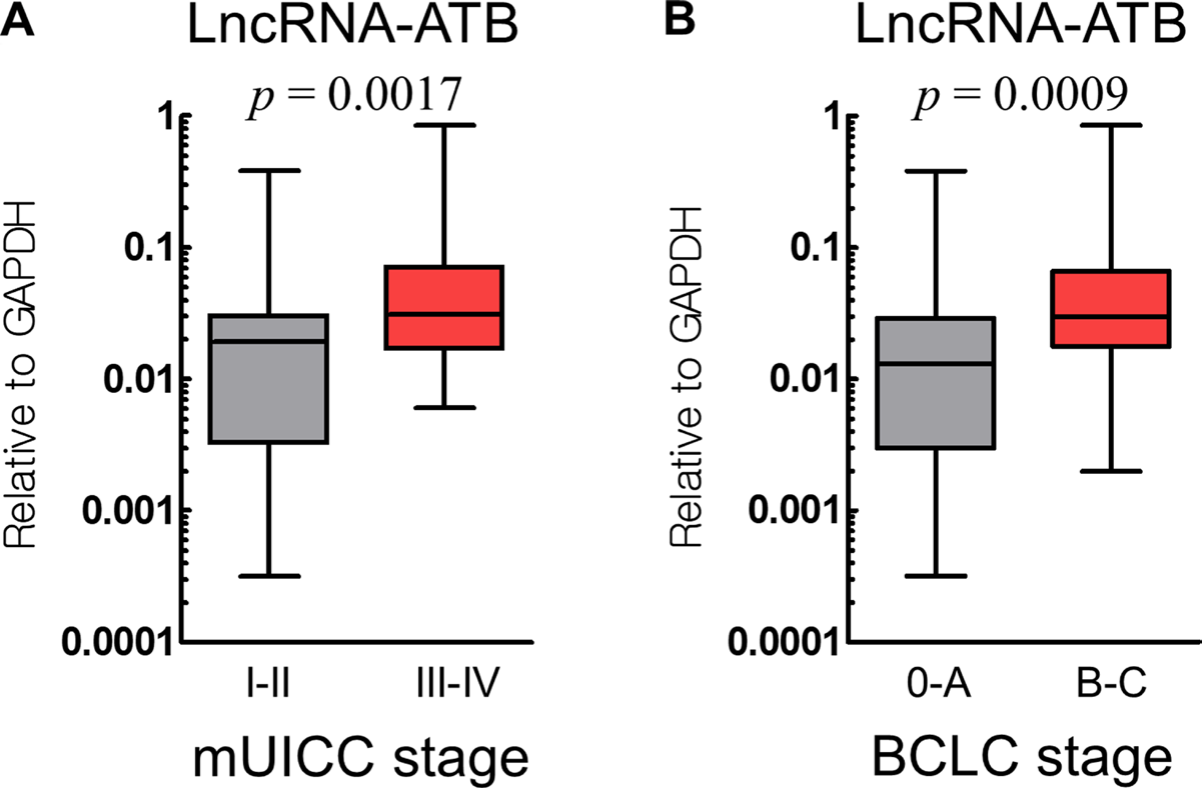
Expression of lncRNA-ATB according to the cancer stage, mUICC stage (**A**) and BCLC stage (**B**).

We analyzed the association between lncRNA-ATB and lncRNA-MEG3 expression and the HCC clinical characteristics (Table 2). Portal vein thrombosis (*p* = 0.028), intrahepatic or extrahepatic metastases (*p* = 0.017), mUICC stage (*p* = 0.011), and the Barcelona Clinic Liver Cancer (BCLC) stage (*p* = 0.002) were clinical factors that correlated with a higher expression of lncRNA-ATB. In contrast, lncRNA-MEG3 expression did not show any correlation with these clinical factors. Figure 2 shows how lncRNA-ATB expression distributed according to the cancer stages (mUICC and BCLC).

**Table 2:** Baseline characteristics of the enrolled HCC patients according to lncRNA-ATB expression

Clinical characteristics	LncRNA-ATB expression			LncRNA-MEG3 expression		
	Low (*n =* 25)	High (*n =* 50)	*p*-value	Low (*n =* 66)	High (*n =* 21)	*p*-value
Age (years)			0.219			0.412
≤ 60	10 (41.7%)	29 (56.9%)		32 (48.5%)	13 (61.9%)	
> 60	14 (58.3%)	22 (43.1%)		34 (51.5%)	8 (38.1%)	
Gender			0.158^a^			1.000
male	18 (75.0%)	46 (90.2%)		58 (87.9%)	18 (85.7%)	
female	6 (25.0%)	5 (9.8%)		8 (12.1%)	3 (14.3%)	
CTP class			0.531^a^			1.000
A	21 (87.5%)	41 (80.4%)		54 (81.8%)	17 (81.0%)	
B	3 (12.5%)	10 (19.6%)		12 (18.2%)	4 (19.0%)	
Tumor number			0.797			0.952
single	13 (54.2%)	26 (51.0%)		32 (48.5%)	11 (52.4%)	
multiple	11 (45.8%)	25 (49.0%)		34 (51.5%)	10 (47.6%)	
Tumor size(cm)			0.076			1.000
≤ 5	17 (70.8%)	25 (49.0%)		35 (53.0%)	11 (52.4%)	
> 5	7 (29.2%)	26 (51.0%)		31 (47.0%)	10 (47.6%)	
PVT			0.028^*^			0.223
no	21 (87.5%)	32 (62.7%)		49 (74.2%)	12 (57.1%)	
yes	3 (12.5%)	19 (37.3%)		17 (25.8%)	9 (42.9%)	
Metastasis			0.017^*^			0.142
no	17 (70.8%)	21 (41.2%)		48 (72.7%)	11 (52.4%)	
yes	7 (29.2%)	30 (58.8%)		18 (27.3%)	10 (47.6%)	
mUICC stage			0.011^*^			0.645
I–II	16 (66.7%)	18 (35.3%)		31 (47.0%)	8 (38.1%)	
III–IV	8 (33.3%)	33 (64.7%)		35 (53.0%)	13 (61.9%)	
BCLC stage			0.002^*^			0.726
O–A	15 (62.5%)	13 (25.5%)		27 (40.9%)	7 (33.4%)	
B–C	9 (37.5%)	38 (74.5%)		39 (59.1%)	14 (66.6%)	
AFP (ng/mL)			0.205			0.223
≤ 200	19 (79.2%)	33 (64.7%)		49 (74.2%)	12 (57.1%)	
> 200	5 (20.8%)	18 (35.3%)		17 (25.8%)	9 (42.9%)	

Note : PVT, portal vein thrombosis

^a^ : Fischer’s exact test

^*^p < 0.05.

### Correlation of lncRNA-ATB expression and survival of HCC patients

Because lncRNA-MEG3 did not show any correlation with the clinicopathological factors, we focused only on lncRNA-ATB for further analysis of the prognostic clinical factors affecting the overall survival of 75 HCC patients. These patients had surgical resection (*n* = 19), radiofrequency ablation (*n* = 22), transarterial chemoembolization (*n* = 5), Sorafenib treatment (*n* = 10), and best supportive care (*n* = 19). Death events occurred in one case out of 12 cases in stage I, three out of 22 in stage II, six out of 15 in stage III, and 21 out of 26 in stage IV. Progression events occurred in four cases out of 12 cases in stage I, nine out of 22 in stage II, nine out of 15 in stage III, and 24 out of 26 in stage IV.

In the univariate analysis, large tumors (> 5 cm, hazard ratio (HR) = 5.320, 95% confidence interval (CI) = 2.360–11.970, *p* < 0.001), poor CTP class (CTP B, HR = 2.510, 95% CI = 1.120–5.650, *p* = 0.025), higher lncRNA-ATB expression (HR = 5.530, 95% CI = 1.680–18.230, *p* = 0.002), and higher AFP level (> 200 mg/dL, HR = 3.070, 95% CI = 1.510–6.230, *p* = 0.005) were significant prognostic factors for overall survival. In the multivariate analysis, large tumors (> 5 cm, HR = 3.851, 95% CI = 1.431–10.364, *p* = 0.008) and higher lncRNA-ATB expression (HR = 4.158, 95% CI = 1.226–14.107, *p* = 0.022) were the prognostic factors for overall survival (Table 3).

**Table 3:** Univariate and multivariate analyses of clinical factors and lncRNA-ATB expression associated with overall survival

	Univariate analysis		Multivariate analysis	
	Hazard ratio (95% CI)	*p*-value	Hazard ratio (95% CI)	*p*-value
Age (> 60 years)	0.860 (0.420–1.740)	0.673		
Number of tumors (multiple vs. single)	0.900 (0.440–1.820)	0.767		
Size of tumors (> 5 cm)	5.320 (2.360–11.970)	< 0.001	3.851 (1.431–10.364)	0.008^*^
CTP class	2.510 (1.120–5.650)	0.025^*^	1.443 (0.618–3.367)	0.396
LncRNA-ATB (high vs. low)	5.530 (1.680–18.230)	0.002^*^	4.158 (1.226–14.107)	0.022^*^
AFP (> 200 ng/mL)	3.070 (1.510–6.230)	0.005^*^	1.185 (0.506–2.778)	0.696

Note: CI, confidence interval

^*^ *p* < 0.05.

**Figure 3:**
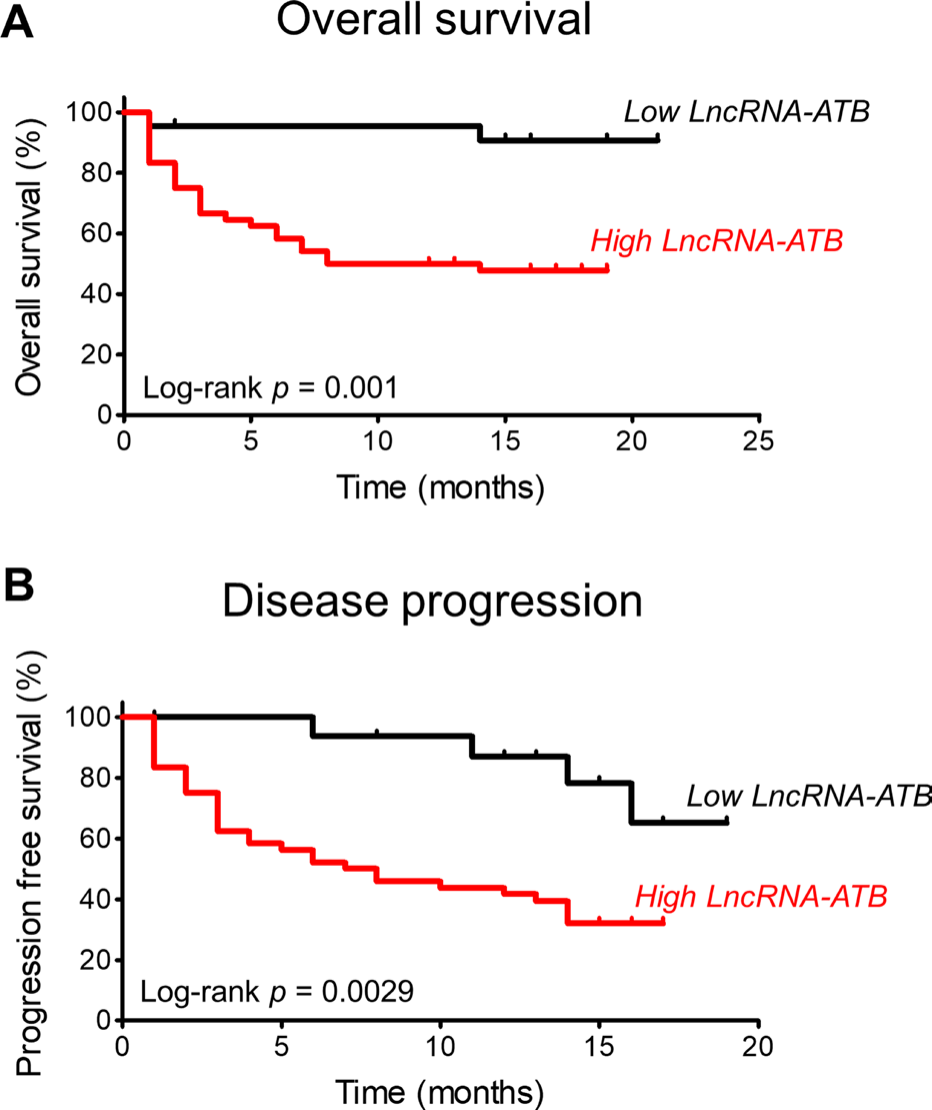
Kaplan-Meier analyses of the correlations between lncRNA-ATB expression level and overall survival (**A**) and progression-free survival (**B**).

The overall survival of patients was significantly different according to the level of expression of lncRNA-ATB (Figure 3A).

### Correlation of lncRNA-ATB expression with progression-free survival of HCC patients

We next analyzed the prognostic clinical factors affecting the progression-free survival of HCC patients (Table 4). In the univariate analysis, large tumors (> 5 cm, HR = 5.030, 95% CI = 2.550–9.920, *p* < 0.001), poor CTP class (HR = 2.110, 95% CI = 1.000–4.440, *p* = 0.005), higher lncRNA-ATB expression (HR = 3.690, 95% CI = 1.440–9.920, *p* < 0.001), and higher AFP level (> 200 mg/dL, HR = 2.300, 95% CI = 1.210–4.380, *p* = 0.012) were significant prognostic factors for progression-free survival. In the multivariate analysis, large tumors (> 5 cm, HR = 5.704, 95% CI = 2.303–14.128, *p* < 0.001) and higher lncRNA-ATB expression (HR = 2.971, 95% CI = 1.121–7.872, *p* = 0.029) were the prognostic factors for progression-free survival.

**Table 4:** Univariate and multivariate analyses of clinical factors and lncRNA-ATB expression associated with progression-free survival

	Univariate analysis		Multivariate analysis	
	Hazard ratio (95% CI)	*p*-value	Hazard ratio (95% CI)	*p*-value
Age (> 60 years)	0.830 (0.440–1.550)	0.554		
Number of tumors (multiple vs. single)	1.010 (0.540–1.870)	0.987		
Size of tumors (> 5 cm)	5.030 (2.550–9.920)	< 0.001^*^	5.704 (2.303–14.128)	< 0.001^*^
CTP class	2.110 (1.000–4.440)	0.005^*^	0.963 (0.416–2.228)	0.930
LncRNA-ATB (high vs. low)	3.690 (1.440–9.920)	< 0.001^*^	2.971 (1.121–7.872)	0.029^*^
AFP (> 200 ng/mL)	2.300 (1.210–4.380)	0.012^*^	0.619 (0.270–1.419)	0.257

Note: CI, confidence interval

^*^ *p* < 0.05.

Similar to the overall survival, progression-free survival was significantly different according to the level of expression of lncRNA-ATB (Figure 3B).

## DISCUSSION

The aim of this study was to determine the clinical relevance of the expression of lncRNAs in HCC. We found associations of lncRNA-ATB with clinicopathological features and its expression was increased in HCC tissues. To the best of our knowledge, this is the first report to validate the clinical significance of lncRNA-ATB in HCC patients ever since the function of this lncRNA in EMT in HCC was reported [13].

LncRNA-ATB, which is activated by TGF-β, has been reported in many studies relating to other varieties of cancers [11, 12, 25–32] and diseases [33, 34]. It was first identified in HCC metastases. TGF-β signaling has an important role in suppressing the growth of normal epithelial cells, while it promotes metastasis in many tumor cells by tightly controlling the EMT process [35]. TGF-β-activated lncRNA-ATB upregulates ZEB1 and 2 by competitively binding to miR-200 family members [12, 13]. It is also known to suppress E-cadherin, a tumor suppressor gene that inhibits the progression of epithelial tumor cells via EMT suppression [13, 27]. In addition, lncRNA-ATB promotes organ colonization of disseminated tumor cells by binding to interleukin-11 mRNA and triggering the STAT3 signaling pathway [13, 36]. LncRNA-ATB promotes cancer cell invasion by inducing the EMT process, which is widely known as a mechanism controlling early events during the metastatic dissemination of cancer cells.

Ever since lncRNA-ATB was first discovered to be involved in HCC metastasis, there have been few studies about the association of its expression with HCC clinical data. Therefore, we analyzed the clinical relevance of lncRNA-ATB in HCC patients. Our results confirmed that lncRNA-ATB is upregulated in HCC tissues compared with its levels in matched, adjacent, non-cancerous liver tissues. Moreover, it is upregulated more in HCCs with portal vein thrombosis and intrahepatic or extrahepatic metastases than in HCCs without those characteristics. These results verified the correlation of lncRNA-ATB expression with HCC progression and metastasis. LncRNA-ATB levels also turned out to be a significant prognostic factor for overall survival and progression-free survival of afflicted patients. An expression level of lncRNA-ATB for discriminating between death and survival was determined by the cutoff value from the receiver operating characteristic curve. The sensitivity and specificity of lncRNA-ATB expression for the prediction of death were 90.3% and 50.0%, respectively, and for the prediction of progression were 81.4% and 53.1%, respectively.

However, there were some limitations to this study. First, there were a few instances of selection bias. Because there were insufficient amounts of some HCC samples to analyze both lncRNA-ATB and lncRNA-MEG3 and, furthermore, some clinical data of patients were missing before their specimens were obtained, we excluded those cases. Therefore, 75 cases were finally analyzed for lncRNA-ATB and 87 cases for lncRNA-MEG3. Moreover, biopsy or surgical resection can be done in patients who preserve better liver function, such as those with CTP class A or B, which also results in selection bias. Second, because tissue samples were obtained mostly by percutaneous liver biopsy, it was impossible to check for microvascular invasion or the histological grade of an entire sample. Pathological features, such as microvascular invasion, histological grade, lymphatic invasion, or cell type, are known to be important factors affecting survival in HCC. In addition, in the case of advanced large tumors, a needle biopsy cannot reflect tumor heterogeneity. Third, study samples were relatively small and the follow-up period was not enough to observe the long-term outcome of the patients. Since lncRNA-ATB expression, treatment modalities, and cancer stage can all affect survival and progression, we analyzed overall survival and progression-free survival in subgroups that had the same cancer stage or received the same treatment according to the expression of lncRNA-ATB. However, results showed no statistical differences between high-expression and low-expression patients. This is because stage IV patients have higher expression of lncRNA-ATB and almost all patients died or progressed during the period. More study samples are needed to overcome this limitation. In contrast, during the follow-up period, death or progression rarely occurred in stage I and II HCC patients. The study period was too short to analyze the overall and progression-free survivals of stage I and II patients. Further investigation with more study patients and a longer observation period is needed. Fourth, this study did not show the function or mechanism of lncRNA-ATB’s effect on clinical characteristics, such as metastasis and cancer progression. Further, *in vitro* or *in vivo* studies are needed to elucidate the mechanism of action of this lncRNA. Fifth, seven patients received liver transplants during the follow-up period, which could have had a positive effect on their survival.

Nevertheless, the results of this study definitively showed that lncRNA-ATB is clinically involved in HCC metastasis and invasion. Moreover, lncRNA-ATB is an independent factor for HCC survival and disease progression and can thus be a potential prognostic biomarker and therapeutic target in HCC. Further study is needed to elucidate the mechanism of lncRNA-ATB action in HCC progression and metastasis and to develop effective individual therapeutic strategies for HCC patients to improve their clinical outcomes.

## MATERIALS AND METHODS

### Patients and clinical specimens

We enrolled HCC patients, at Kyungpook National University Hospital, Republic of Korea from March 2015 to August 2015, who underwent liver biopsy or surgical resection and were in Child-Pugh class A or B for liver function. Diagnostic biopsy was performed for definite diagnosis to exclude other liver malignancies and surgical resection was performed for curative treatment of HCC. Liver biopsies were done in 56 (56%) patients and liver resections in 44 (44%) patients. We excluded patients who no longer received follow-up or had received previous therapies for HCC. We excluded patients diagnosed with other liver malignancies and included patients diagnosed with HCC by pathological examination.

For surveillance of HCC after treatment, patients received a contrast-enhanced dynamic computed tomography (CT) scan or liver magnetic resonance imaging (MRI) every 3 to 6 months after HCC treatment. Overall survival was measured from the date of diagnosis until death from any cause and progression-free survival was measured from the date of diagnosis to the first recurrence, progression, or to death from any cause. HCC recurrence was defined as the presence of a tumor with a size ≥1 cm with typical findings in contrast enhancement in the arterial phase, followed by contrast washout in the venous or delayed phase of CT or MRI. Tumor response was assessed by Response Evaluation Criteria in Solid Tumors (RECIST) version 1.1.

Tumor tissues and matched adjacent non-tumor tissues were obtained, with informed consent, from 100 HCC patients. All samples were stored immediately in RNAlater solution (Ambion; Life Technologies, Carlsbad, CA, USA) at 4°C for 24 h, after which the RNAlater solution was removed and the samples were stored at –80 °C. All clinicopathological data, including age, sex, the number of tumors, the sizes of tumor, the presence of portal vein thrombosis, mUICC stage, BCLC stage, AFP, intrahepatic or extrahepatic metastasis, CTP class, and laboratory findings, were recorded. This study was approved by the Institutional Review Board of Kyungpook National University Hospital (KNUH-2014-04-056-001).

### RNA isolation

Total RNA was extracted from the clinical tissue samples using the QIAzol Lysis Reagent (Qiagen, Valencia, CA, USA) according to the manufacturer’s instructions. The quantity and quality of the isolated total RNA were determined using a NanoDrop ND-1000 spectrophotometer (Thermo Fischer Scientific, Wilmington, DE, USA).

### Quantitative reverse-transcription polymerase chain reaction

Reverse-transcription reactions were carried out using High-Capacity cDNA Reverse Transcription Kits (Applied Biosystems, San Diego, CA, USA). The expression levels of the two lncRNAs were measured by the quantitative reverse-transcription polymerase chain reaction (qRT-PCR) using the Power SYBR Green Master Mix (Applied Biosystems). The qRT-PCR analyses were performed with the QuantStudio 6 Flex Real-Time PCR System (Applied Biosystems), using the following primers: sense-lncRNA-ATB (5′-TCTGGCTGAGGCTGGTTGAC-3′) and antisense-lncRNA-ATB (5′-ATCTCTGGGTGCTGGTGAAGG-3′); sense-lncRNA-MEG3 (5’-GCATTAAGCCCTGACCTTTG-3’) and antisense-lncRNA-MEG3, (5’-TCCAGTTTGCTAGCAGGTGA-3’); and sense-GAPDH (5′-GGAAGGTGAAGGTCGGAGTC-3′) and antisense-GAPDH (5′-GTTGAGGTCAATGAAGGGGTC-3′). Cycle threshold (Ct) values were calculated by using the same threshold cut-off values for each assay to prevent plate-to-plate variations. The relative expression was calculated using the 2^−ΔΔCt^ method. Each experiment was performed in triplicates.

### Statistical analysis

Statistical analysis was performed using SAS software (version 9.4, SAS Institute Inc., Cary, NC, USA) and GraphPad Prism (version 6.0 for Windows, GraphPad Software, La Jolla, CA, USA). Data are presented as the number (%), mean ± standard deviation, or 25–75% interquartile range. The chi-squared or Fisher exact tests were used to compare differences in clinical characteristics between two groups according to the expression of lncRNA-ATB. Survival curves were calculated using the Kaplan-Meier method, and differences between the curves were analyzed using the log-rank test. A Cox proportional hazards model was used for the multivariate survival analyses. Statistical significance was defined as a *p-*value of < 0.05.

## Abbreviations

HCC: hepatocellular carcinoma
AFP: alpha-fetoprotein
lncRNA: long non-coding RNA
lncRNA-ATB: lncRNA activated by TGF-β
lncRNA-MEG3: lncRNA-maternally expressed gene 3
EMT: epithelial mesenchymal transition
mUICC: modified Union for International Cancer Control
BCLC: Barcelona Clinic Liver Cancer
CTP: Child-Turcotte-Pugh
qRT-PCR: quantitative reverse-transcription polymerase chain reaction
